# Chronotropic and vasoactive properties of the gut bacterial short-chain fatty acids in larval zebrafish

**DOI:** 10.1152/physiolgenomics.00013.2024

**Published:** 2024-04-01

**Authors:** Hemaa Sree Kumar, Alexander S. Wisner, Isaac T. Schiefer, Adriana Alviter Plata, Jasenka Zubcevic

**Affiliations:** ^1^Department of Physiology and Pharmacology, University of Toledo College of Medicine and Life Sciences, Toledo, Ohio, United States; ^2^Department of Neurosciences, University of Toledo College of Medicine and Life Sciences, Toledo, Ohio, United States; ^3^Department of Medicinal and Biological Chemistry, University of Toledo College of Pharmacy and Pharmaceutical Sciences, Toledo, Ohio, United States; ^4^Center for Drug Design and Development, University of Toledo College of Pharmacy and Pharmaceutical Sciences, Toledo, Ohio, United States

**Keywords:** acetate, butyrate, cardiovascular, microbiota, zebrafish

## Abstract

Short-chain fatty acids (SCFAs) produced by the gut bacteria have been associated with cardiovascular dysfunction in humans and rodents. However, studies exploring effects of SCFAs on cardiovascular parameters in the zebrafish, an increasingly popular model in cardiovascular research, remain limited. Here, we performed fecal bacterial 16S sequencing and gas chromatography/mass spectrometry (GC-MS) to determine the composition and abundance of gut microbiota and SCFAs in adult zebrafish. Following this, the acute effects of major SCFAs on heart rate and vascular tone were measured in anesthetized zebrafish larvae using fecal concentrations of butyrate, acetate, and propionate. Finally, we investigated if coincubation with butyrate may lessen the effects of angiotensin II (ANG II) and phenylephrine (PE) on vascular tone in anesthetized zebrafish larvae. We found that the abundance in *Proteobacteria*, *Firmicutes*, and *Fusobacteria* phyla in the adult zebrafish resembled those reported in rodents and humans. SCFA levels with highest concentration of acetate (27.43 µM), followed by butyrate (2.19 µM) and propionate (1.65 µM) were observed in the fecal samples of adult zebrafish. Immersion in butyrate and acetate produced a ∼20% decrease in heart rate (HR), respectively, with no observed effects of propionate. Butyrate alone also produced an ∼25% decrease in the cross-sectional width of the dorsal aorta (DA) at 60 min (*P < 0.05), suggesting compensatory vasoconstriction, with no effects of either acetate or propionate. In addition, butyrate significantly alleviated the decrease in DA cross-sectional width produced by both ANG II and PE. We demonstrate the potential for zebrafish in investigation of host-microbiota interactions in cardiovascular health.

**NEW & NOTEWORTHY** We highlight the presence of a core gut microbiota and demonstrate in vivo short-chain fatty acid production in adult zebrafish. In addition, we show cardio-beneficial vasoactive and chronotropic properties of butyrate, and chronotropic properties of acetate in anesthetized zebrafish larvae.

## Graphical abstract

**Figure:**
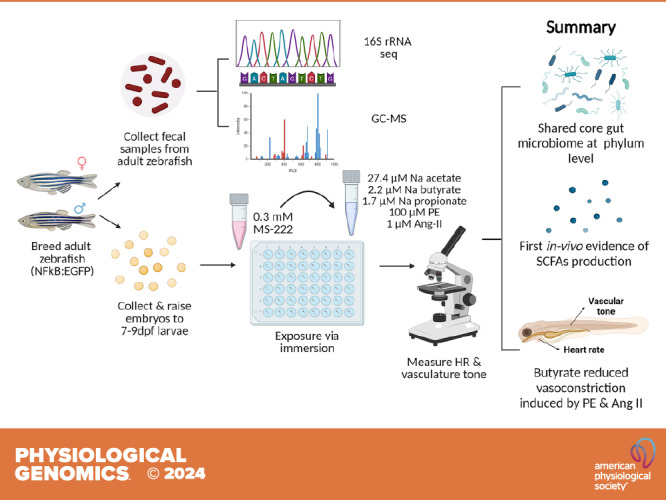

## INTRODUCTION

Zebrafish (*Danio rerio*), a teleost model and a member of the *Cyprinidae* family, has become an increasingly popular vertebrate model in biomedical research due to advantages that include genome homology, optical clarity, high fecundity and rapid development, high throughput capability, ease of genetic manipulation, and cost efficiency in husbandry (1–3). In addition to these, it is also considered a valuable model in cardiovascular research (4), and a variety of techniques are currently available for physiological hemodynamic measurements in adults and larval zebrafish (5–19).

The gut bacteria produce short-chain fatty acids (SCFAs) by fermentation of resistant starch and dietary fibers in the colon (20–22). Acetate, propionate, and butyrate are three major SCFA metabolites produced in the colon with approximate molar ratio of 3:1:1 in humans (20, 21) and 8:1:1 in rodents (23), but their physiological composition in the zebrafish is unknown. SCFAs can modify host physiology via anti-inflammatory, cardio-, hepato-, and neuro-protective effects (22, 24–26). These include regulation of blood pressure (24, 26–29); however, how the SCFAs may affect the cardiovascular system in the zebrafish is unknown.

Although the composition of the zebrafish microbiota has been reported (30, 31), and the ability of the zebrafish gut bacteria to synthesize three major SCFAs has been demonstrated in vitro (32), to the best of our knowledge, there have been no studies confirming zebrafish SCFAs production in vivo. Studies exploring the effects of SCFAs on cardiovascular parameters in the zebrafish are also lacking. Here, we took advantage of the optical clarity of the zebrafish larvae to investigate the effects of major SCFAs on the heart and vascular tone in vivo. We report similarities in the gut microbiota and SCFA composition and concentration in the zebrafish with that of rodent models and humans. This, coupled with the observed cardiovascular effects of select SCFAs, and the high throughput capability of the zebrafish larvae, suggests a use for zebrafish in understanding host-microbiota interactions in cardiovascular health.

## MATERIALS AND METHODS

### Animals and Husbandry

Adult Tg(NFkB:EGFP) *D. rerio* zebrafish were purchased from Zebrafish International Resource Center (Eugene, OR), bred and raised inhouse at the University of Toledo Center for Drug Design and Development Zebrafish Core Facility on a recirculating water system (Tecniplast, 28°C, 14:10-h light:dark cycle). Adult zebrafish were fed live brine shrimp (*Artemia salina;* 58% crude protein, 4.4% crude fat, 7% crude fiber), and supplemented by Golden Pearl Spheres [300–500 μm, Brine Shrimp Direct; 55% protein, 15% lipids, 12% ash, 2,550 ppm vitamin C, 425 ppm vitamin E, 10 mg/g eicosapentaenoic acid (EPA), and 12 mg/g docosahexaenoic acid (DHA)] once daily. To generate the larvae, male and female adult zebrafish (8–12 mo) were placed together in a breeding tank (1:2 ratio of male to female) overnight. Embryos were collected the following morning and raised in embryo water (sterile distilled water with adjusted salinity, pH 7.6, at 28 ± 1°C). Following experimental procedures, all subjects were euthanized by rapid chilling by submerging in 2–4°C ice-chilled water for 30 min, and death was confirmed by observation of cessation of opercular movement. All experiments and husbandry practices were performed in accordance with the Institutional Animal Care and Use Committee guidelines at the University of Toledo (IACUC No. 400140 and 400099).

### Gut Bacterial and Short-Chain Fatty Acid Analyses

For collection of fecal samples, adult zebrafish (*n* = 10–12/tank/sex, ∼15 mo) were placed in a 1.7-L sloped breeding tank with a perforated internal tank overnight. Fecal samples (100 mg/sample) were collected the following morning and centrifuged (2,000 rpm, 10 min). Excess water was removed from fecal samples using a suction micropipette following centrifugation. Three tanks contained males and the other three tanks were females (*n* = 10–12 subjects/tank/sample, defined as *n* = 6 pooled samples). Analysis was done independently of sex. Water sample from the housing tank was used as a control to confirm no contamination. Once collected, samples were stored at −80°C until further processing. For bacterial 16S rRNA gene sequencing, DNA extraction was performed using QIAam PowerFecal DNA kit (Qiagen) as previously described (33). Bacterial 16S rRNA gene sequencing was performed on an Illumina MiSeq platform as previously described (33) and raw 16S data were analyzed using Quantitative Insights Into Microbial Ecology (QIIME2, v.2023.9) and SILVA database (v.132) (34–36). Data from QIIME2 were further filtered to remove amplicon sequence variants (features) with low abundance (<1%) after summing across all samples in RStudio (v.2023.09.0 + 463) for descriptive analyses and presented as bar plots. In the same fecal samples, SCFA analysis was performed by gas chromatography mass spectrometry (GC-MS) (Creative Proteomics, Shirley, NY) (29, 37). Briefly, fecal samples were diluted in water with labeled internal standards for each chain length (C2–C6). The free SCFAs were derivatized using methyl chloroformate in 1-propanol yielding propyl esters before subsequent liquid-liquid extraction into hexane and analysis on a SLB-5ms (30 × 0.25 mm × 1.0 μm) column. Detection was performed using gas chromatography electron ionization mass spectrometry (GC-EI-MS) in selective ion monitoring (SIM) mode and compared with standards. The analytes were quantified using a six-point calibration curves. Final SCFA levels were calculated as µg per g of wet feces.

### Effect of Short-Chain Fatty Acids on Heart Rate and Vascular Tone in the Zebrafish Larvae

All experimental procedures used zebrafish larvae that exhibited normal developmental endpoints at 7–9 days postfertilization (dpf) (38, 39). Zebrafish larvae (*n* = 5–6/treatment) were anesthetized with tricaine (MS-222, Sigma Aldrich; Cat. No. E10521) by immersion (0.3 mM, pH 7.6) for 10 min before measurements. The appropriate tricaine dosage was established in preliminary experiments showing no effects on heart rate (HR) as per Ref. 40. Following baseline measurements, subjects were immersed in embryo water containing either sodium butyrate (2.2 µM, pH 7.6; Sigma-Aldrich; Cat. No. B5887), sodium acetate (27.4 µM, pH 7.6; Sigma-Aldrich; Cat. No. S2889), or sodium propionate (1.7 µM, pH 7.6; Sigma-Aldrich; Cat. No. P1880). These concentrations were determined using GC-MS in zebrafish fecal samples, and final dosages for administration were based on estimated fecal weight (1% of average body weight of the zebrafish larvae) and expressed as µM (41). All compounds were dissolved in embryo water and the pH was adjusted prior to administration. For measurements of effects of SCFAs on HR and vascular tone, larvae were positioned dorsally to allow for optical clarity, and heartbeat was recorded for 10 s every 10 min for an hour and averaged to beats per minute (BPM) in real time under a stereo microscope (Zeiss Stemi 2000-C). To measure effects of SCFA on vascular tone, in anesthetized zebrafish larvae (*n* = 6–8/treatment), baseline video recordings (representing 0 min timepoint) of dorsal aorta (DA) were taken for 30 s under a phase contrast microscope (Nikon SMZ18, 4× magnification with 1.6× lens). Following administration of SCFAs to the wells containing the larvae, video recordings of the same DA region were taken for 30 s every 10 min for 1 h. For analysis, still images of recordings were exported into ImageJ (v.1.54d). DA cross-sectional width for each treatment was determined by measurements at 10 different points along DA length and averaged for each treatment, timepoint, and larvae.

### Effect of Phenylephrine and Angiotensin II on Heart Rate and Vascular Tone in the Zebrafish Larvae

Zebrafish larvae (*n* = 5–6/treatment) were anesthetized with tricaine by immersion for 10 min before baseline measurements, as previously described. To measure the effects of angiotensin acetate salt II (ANG II) and phenylephrine (PE) on HR, compounds (100 µM of PE, pH 7.6; Sigma-Aldrich; Cat. No. P1240000, or 1 µM of ANG II, pH 7.6; Bachem; Cat. No. 4006473 in embryo water) were administered in separate well plates containing anesthetized larvae. Concentrations of ANG II and PE were based on previous reports of their vasoconstrictive effects (42, 43). HR and vascular tone were imaged as described previously, before and following administration of ANG II or PE to the wells, every 10 min for an hour. Analysis of images was performed as described earlier.

### Coincubation of Phenylephrine and Angiotensin II with Sodium Butyrate

Zebrafish larvae (*n* = 5–10/treatment group) were anesthetized with tricaine by immersion for 10 min before treatments, as previously described. Following baseline measurements, zebrafish were administered with 100 µM of PE, 1 µM of ANG II, and 2.2 µM of sodium butyrate alone or in combination with PE or ANG II. Changes in the vascular tone were analyzed as described previously.

### Statistical Analyses

16S rRNA sequencing data was analyzed using QIIME2 (v.2023.9) and SILVA database (v.132) (34–36). GraphPad Prism (v.10.0.3) was used for all other statistical analysis. One-way ANOVA was used to analyze parametric end points followed by Dunnett’s multiple comparison test. Repeated-measures ANOVA was used when variables are assessed over time in the same subjects followed by Dunnett’s multiple comparison test. Two-way ANOVA was used to measure interaction between two independent variables on the dependent variable followed by appropriate post hoc test. Sample size for each treatment group and statistical test used are denoted in the figures. All data are presented as means ± SE.

## RESULTS

### 16S and SCFA Analyses Reveal the Prevalence of Three Major Phyla and SCFAs

**Figure 1.**
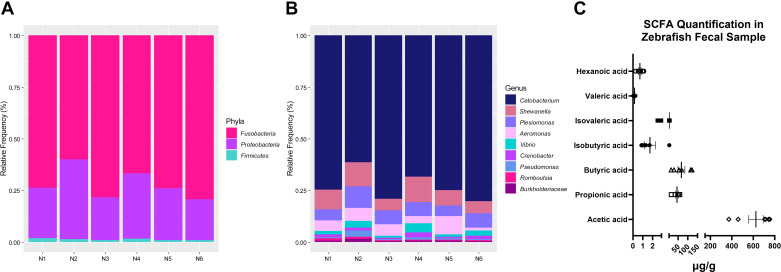
Relative abundance of bacterial taxa and composition of short-chain fatty acids (SCFAs) in adult zebrafish. *A* and *B*: 16S rRNA sequencing showing three major phyla (*A*) and nine genera (*B*) that make up the gut microbiota in the adult zebrafish (*n* = 6 pooled samples from 10 to 12 zebrafish/tank/sample). Bar plots show the relative abundance of bacterial taxa in each pooled sample created in RStudio. *C*: SCFA composition and abundance in fecal samples of adult zebrafish (*n* = 6 pooled samples from 10 to 12 zebrafish/tank/sample).

We observed three major phyla in fecal samples of adult zebrafish, namely, the *Fusobacteria*, *Proteobacteria*, and *Firmicutes* (Fig. 1*A*) that make up the gut microbiota. At the genus level, there was an abundance of *Cetobacterium*, followed by *Shewanella*, *Plesiomonas*, *Aeromonas*, *Vibrio*, *Crenobacter*, *Pseudomonas*, *Romboutsia*, and, finally, *Burkholderiaceae* (Fig. 1*B*). GC-MS analysis revealed the highest concentration of acetate, followed by butyrate, propionate, isovalerate, isobutyrate, hexanoate, and valerate (Fig. 1*C*).

### Butyrate and Acetate Induced Bradycardia With No Major Effects on Vascular Tone

**Figure 2.**
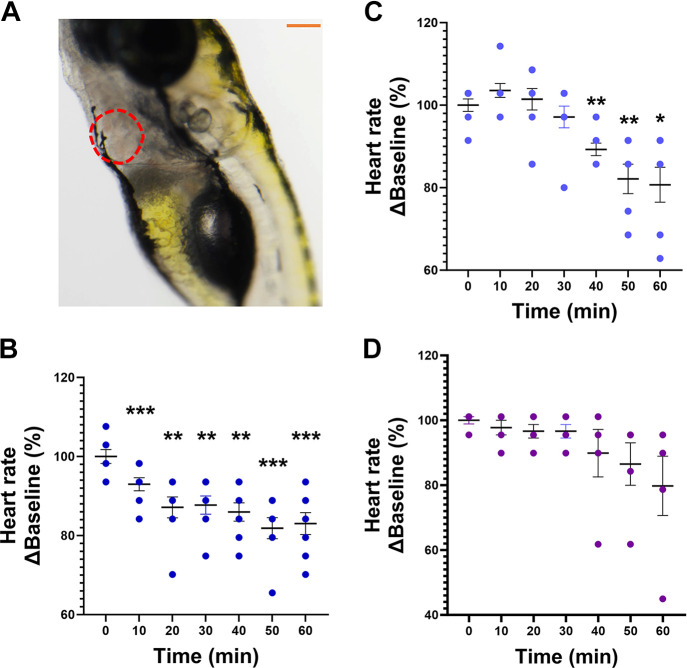
Effects of sodium butyrate, sodium acetate, and sodium propionate on heart rate (HR) in anesthetized zebrafish larvae. *A*: representative image showing optical clarity allowing for visualization of the heart (in red dotted lines). Scale bar in orange = 200 µm. *B−D*: effects of administration of 2.2 µM butyrate (*B*), 27.4 µM acetate (*C*), and 1.7 µM propionate (*D*) on HR in zebrafish larvae, calculated as %Baseline (0 min). Data are presented as means ± SE (*n* = 5–8/treatment group). One-way ANOVA with a Dunnett’s post hoc test; *P < 0.05, **P < 0.01, ***P < 0.001.

**Figure 3.**
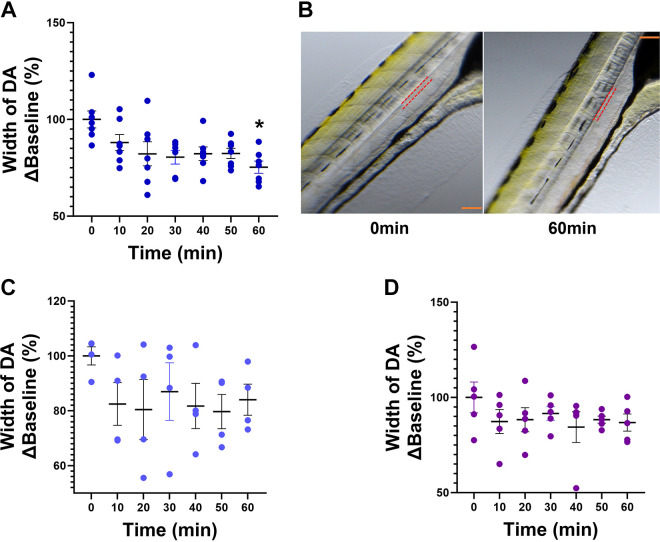
Effects of sodium butyrate, sodium acetate, and sodium propionate on vascular tone in anesthetized zebrafish larvae. *A*: 2.2 µM sodium butyrate produced a significant decrease in the cross-sectional width of the dorsal aorta (DA) at 60 min. *B*: red dotted lines mark the outline of the DA at baseline (0 min, *left*) and at 60 min (*right*) following exposure to sodium butyrate. Scale bar in orange = 200 µm. *C* and *D*: 27.4 µM acetate (*C*) and 1.7 µM propionate (*D*) produced no change. Data are presented as means ± SE and calculated as %Baseline (0 min). *n* = 5–8/treatment group. Repeated-measures one-way ANOVA with a Dunnett’s post hoc test; *P < 0.05.

Figure 2*A* illustrates the optical clarity of zebrafish larvae that allows visualization of the heart under a light microscope. Exposure to sodium butyrate (2.2 µM, *n* = 8) (Fig. 2*B*) reduced HR at all time points (**P < 0.01, ***P < 0.001), whereas sodium acetate (27.4 µM, *n* = 8) produced a significant decrease in HR at 40 min (**P < 0.01), 50 min (**P < 0.01), and 60 min following immersion (*P < 0.05) (Fig. 2*C*) when compared with baseline (0 min). In contrast, sodium propionate (1.7 µM, *n* = 5) (Fig. 2*D*) produced no effect on HR at any time point. Exposure to sodium butyrate (*n* = 7, *P < 0.05) reduced DA cross-sectional width at 60 min when compared with baseline (0 min) (Fig. 3*A*). Figure 3*B* shows representative images of the DA outlined in red dotted lines at baseline (*left image*) and at 60 min (*right image*) following butyrate treatment. Exposure to sodium acetate (*n* = 4) or propionate (*n* = 5) produced no significant effects on the cross-sectional width of the DA (Fig. 3, *C* and *D*).

### Butyrate Reduced the Effects of PE and ANG II on the Cross-Sectional Width of the Dorsal Aorta

**Figure 4.**
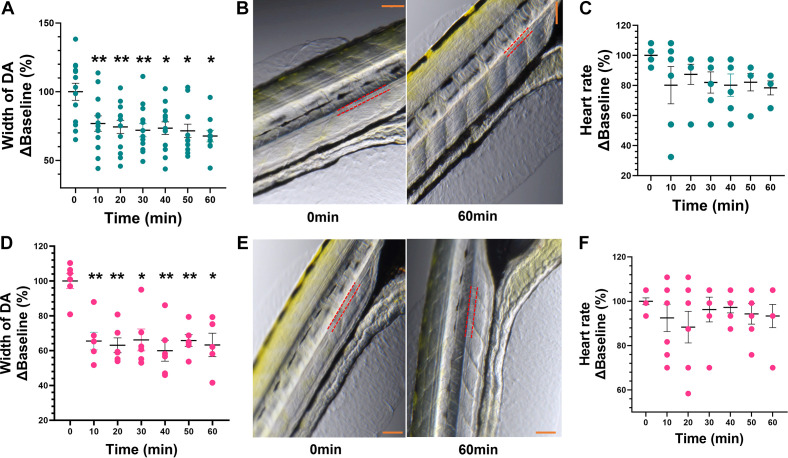
Effects of phenylephrine and angiotensin II (ANG II) on vascular tone and heart rate in anesthetized zebrafish larvae. *A* and *D*: administration of 1 µM ANG II (*A*) and 100 µM phenylephrine (PE) (*D*) produced a decrease in the cross-sectional width of dorsal aorta (DA) at all time points when compared with baseline (0 min). *B* and *E*: representative images at baseline (0 min) and the 60-min timepoint for ANG II (*top*) and PE (*bottom*), with red dotted lines marking the outline of the DA. Scale bar in orange = 200 µm. *C* and *F*: ANG II (*C*) and (*F*) PE had no effect on heart rate (beats/min). Data are presented as means ± SE (*n* = 6–7 PE and 6–13 ANG II) and calculated as %Baseline (0 min). Repeated-measures one-way ANOVA with a Dunnett’s post hoc test; *P < 0.05, **P < 0.01.

**Figure 5.**
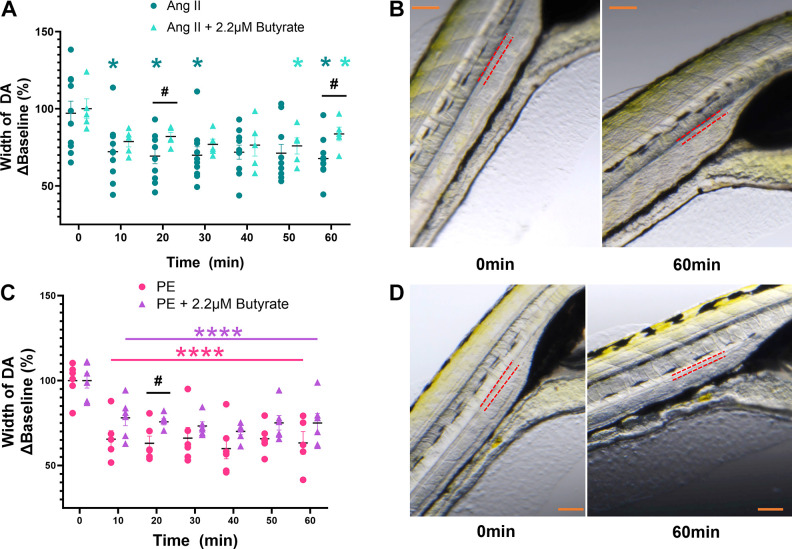
Effect of coadministration of sodium butyrate with phenylephrine (PE) and angiotensin II (ANG II) on vascular tone. *A*: 1 µM ANG II alone (in dark teal) significantly decreased dorsal aorta (DA) cross-sectional width at several time points compared with baseline (0 min). Coadministration of sodium butyrate (in light teal) reduced the effects of ANG II at 20 and 60 min. *B*: representative DA images at 0 and 60 min for ANG II, with red dotted lines marking the outline of the DA. Scale bar in orange = 200 µm. *C*: 100 µM PE alone (in pink) produced a significant decrease in DA cross-sectional width at all timepoints when compared with baseline (0 min). Coadministration of sodium butyrate (in purple) significantly reduced the effects of PE at 20 min. *D*: representative DA images at 0 and 60 min for PE, with red dotted lines marking the outline of the DA. Scale bar in orange = 200 µm. Data are presented as means ± SE and calculated as %Baseline (0 min). *n* = 5–10/treatment group. Two-way ANOVA with a Dunnett’s and Sidak’s multiple comparison test; *P < 0.05, ****P < 0.0001: within-group comparison to baseline (0 min). #*P* < 0.05 comparisons between treatment groups.

Treatments with 1 µM ANG II (*n* = 13) and 100 µM PE (*n* = 6) produced a significant decrease in DA cross-sectional width (∼30%, *P < 0.05, and ∼40%, **P < 0.01, respectively) (Fig. 4, *A* and *D*) with no significant effects on HR (*n* = 6–7/treatment group) when compared with baseline (0 min) (Fig. 4, *C* and *F*). Representative images indicate the outline of the DA with red dotted line where measurements were taken, and white lines show the DA cross-sectional width at 10 different points along the length of DA at baseline and 60 min following exposure to ANG II and PE, respectively (Fig. 4, *B* and *E*). Coadministration of 2.2 µM butyrate with ANG II produced a significant increase (∼10%) in DA cross-sectional width at 20 and 60 min when compared with ANG II alone (Fig. 5*A*) (#*P* < 0.05). In addition, coadministration of 2.2 µM butyrate with PE resulted in ∼15% increase in DA cross-sectional width at 20 min when compared with PE alone (Fig. 5*C*) (#*P* < 0.05). Figure 5, *B* and *D*, shows representative images of the DA at baseline and at 60 min following exposure to ANG II (*top right*) and PE with 2.2 µM butyrate (*bottom right*).

## DISCUSSION

We uncovered several novel findings. We first determined the levels of SCFAs in fecal samples of adult zebrafish in vivo. We found that acetate, followed by butyrate and propionate, were the three major SCFAs present at highest concentrations in adult zebrafish. In humans, the approximate molar ratio of intestinal SCFAs is reportedly 3:1:1 (acetate:propionate:butyrate) (20, 21, 26, 44), and in rodents these ratios are ∼8:1:1 (23, 45). Thus, the relative trend of abundance of the three major SCFAs are similar in zebrafish (16:1:1), rodents, and humans. An approximate 1:1 ratio of propionate:butyrate is found across all three species. In addition, we found that *Fusobacteria*, *Proteobacteria*, and *Firmicutes* were the three most abundant phyla in adult zebrafish, consistent with previous reports (46–48). These phyla are shared with rodents and complex mammals (49). In addition, at the genus level, *Aeromonas* and *Vibrio* are reportedly the two most predominant genera in the zebrafish (50), consistent with our current findings. It is expected that the composition and abundance of the gut bacteria will differ between species owing to many internal and external factors; however, our findings highlight that significant conservation in the gut bacteria and their major SCFA products exists between the zebrafish, rodents, and humans, justifying the use of the zebrafish in investigation of host-microbiota interactions. As SCFAs are metabolic byproducts of dietary fibers and resistant starch, diet can play a role in modifying SCFAs and gut bacterial community. The larval zebrafish rely on the yolk sac for nutrition up to 7 dpf (51), therefore modification of nutrient intake in the young larvae may be challenging. In the adult zebrafish, the dietary nutrients in the feed primarily consist of proteins, with smaller percentages of fat, lipids, fiber, and vitamins. The dietary proteins are crucial for growth and metabolism throughout the zebrafish development (52, 53) making the dietary studies challenging. Despite this, several studies have investigated dietary effects on zebrafish gut microbiota and physiology (9, 54, 55) but studies of zebrafish SCFAs is lacking. The developments in gnotobiotic husbandry in the zebrafish (56–59) provide an accessible avenue for investigation of host-microbiota interactions to researchers limited by the lack of rodent germ-free facilities.

We next investigated the effects of the three most abundant SCFAs (acetate, propionate, and butyrate) on HR and vascular tone in the larvae. We chose to use the larval zebrafish at 7–9 dpf due to their optical clarity at this developmental stage, which enabled us to monitor the HR and vascular effects of SCFAs by light microscopy. Anatomical positioning were aided by study from Fritsche et al. (60), where digital motion analysis investigated the effects of sodium nitroprusside and epinephrine, as well as by the vascular anatomy atlas of the embryonic and early larval development in the zebrafish (61). We chose to focus on the DA as it is the main artery that runs along the trunk and carries oxygenated blood from the gills throughout the body (62, 63). We show that immersion in sodium acetate and butyrate produced a significant bradycardia at several time points within an hour, with no effects of sodium propionate. The effects of sodium butyrate on the heart are consistent with those reported in rats, where activation of GPR41/43 receptors and modulation of the nervous system is reported to play a role (27, 60, 64–67). However, there have not been many studies investigating SCFA receptors in regulation of cardiovascular variables in the zebrafish. One study identified and validated 10 coding sequences for common carp (*Cyprinus carpio*) *gpr40L* gene as it is closely related to the mammalian GPR43 (68). Cholan et al. (32) studied the role of *hcar1* ortholog in reducing inflammatory responses following a tail wound injury in the zebrafish, while a recent study showed evidence of expression of free fatty acid receptor 2 (FFAR2,also known as GPR43) that can be activated by SCFAs in the zebrafish gut-brain axis in cadmium-induced neurodevelopmental toxicity (69). Interestingly, we observed no major effects of SCFAs on the vascular tone when administered alone, contrary to the reports in rodents (70–72) and human colonic arteries (73, 74). We measured the changes in the vascular tone by the increase and decrease in the cross-sectional width of the DA, which were considered as indicators of vasodilation and vasoconstriction respectively, similar to a study by Fritsche et al. (60). A significant decrease in the DA cross-sectional width following 1 h immersion with butyrate, indicative of xpotential vasoconstriction, was attributed to a potential compensatory response to butyrate-induced bradycardia to maintain hemodynamic homeostasis.

Next, we explored whether butyrate may lessen the effects of ANG II and PE, two vasoconstricting agents associated with elevated blood pressure in rodent models of hypertension (75–81). Indeed, we show that both PE and ANG II produced a significant decrease in the cross-sectional width of the DA, suggesting vasoconstrictive properties previously shown in rodents (75–81). The lack of effects of PE and ANG II on zebrafish HR, despite the reported importance of adrenergic receptors and ANG II in regulation of HR in the zebrafish (42, 82–87), may be due to the mode and/or duration of compound delivery in the current study. Interestingly, when coadministered with PE and ANG II, sodium butyrate was able to reduce the vasoconstrictive effects of ANG II and PE but only at select time points. Vasodilating properties of butyrate have been shown in rat mesenteric arteries following vasoconstriction by PE (64); however, to the best of our knowledge, there are no studies reporting vascular effects of butyrate in the zebrafish. Higher doses of butyrate delivered orally may be used in future studies for more pronounced cardiovascular effects.

Our study demonstrates a role for the zebrafish in investigation of host-microbiota interactions in cardiovascular health. We show that the core gut bacterial phyla and major SCFAs are similar between the zebrafish, rodent models, and humans, and that the zebrafish larvae may present as a useful tool in investigation of chronotropic and vasoactive properties of bacterial metabolites associated with cardiovascular health. Furthermore, we demonstrate that butyrate may benefit both the cardiac and vascular function to promote cardiovascular health.

### Limitations

One potential limitation was the use of anesthetizing agent, which can influence cardiovascular measurements. However, tricaine is widely used (40) as an alternative to other immobilization techniques (88), and produced no HR effects in our preliminary measurements. In addition, administration of compounds via immersion relies mostly on passive diffusion and/or absorption, which may have guided the timing and extent of our responses. Moreover, there may be a difference in the concentration of fecal SCFAs between larval and adult zebrafish, as well as between fecal and circulating SCFAs. It is technically challenging to obtain sufficient larval fecal or blood sample or adult zebrafish blood sample (89) for SCFA detection. Thus, we utilized concentrations obtained from adult zebrafish fecal samples for a proof-of-concept experiment in the larvae. Future studies should use SCFA heart and vascular studies in adult zebrafish with direct blood pressure measurements (12, 13) and oral administration of SCFAs to increase specificity.

### Conclusions and Perspectives

Our study provides the first in vivo evidence, to the best of our knowledge, demonstrating the production of SCFAs by the zebrafish gut bacteria similarly to those reported in rodents and humans. We also highlight a shared core gut microbiota between these species. The bradycardic effects of the major SCFAs were consistent with findings reported in rodent models. In addition, our research reveals that butyrate may lessen the PE- and ANG II-induced vasoconstriction, similarly to what has been reported in rodents (64). These findings underscore significant conservation between the zebrafish, rodents, and humans. Coupled with its inherent advantages (90), zebrafish may present a valuable tool in elucidating the intricate interactions of host-microbiota in regulation of cardiovascular health, thus reducing our reliance on complex mammalian systems.

## DATA AVAILABILITY

The data sets generated following 16S rRNA gene sequencing are openly available at https://doi.org/10.6084/m9.figshare.25055612.v2.

## GRANTS

This work was supported by National Institutes of Health Grant HL152162 and University of Toledo startup funds (to J.Z.).

## DISCLOSURES

No conflicts of interest, financial or otherwise, are declared by the authors.

## AUTHOR CONTRIBUTIONS

J.Z. conceived and designed research; H.S.K. performed experiments; H.S.K. and A.A.P. analyzed data; H.S.K. interpreted results of experiments; H.S.K. prepared figures; H.S.K. and J.Z. drafted manuscript; H.S.K., A.S.W., I.T.S., A.A.P., and J.Z. edited and revised manuscript; H.S.K., A.S.W., I.T.S., A.A.P., and J.Z. approved final version of manuscript.
